# Sea urchin vault structure, composition, and differential localization during development

**DOI:** 10.1186/1471-213X-5-3

**Published:** 2005-02-14

**Authors:** Phoebe L Stewart, Miriam Makabi, Jennifer Lang, Carrie Dickey-Sims, Anthony J Robertson, James A Coffman, Kathy A Suprenant

**Affiliations:** 1 Department of Molecular Physiology and Biophysics, Vanderbilt University Medical Center, Nashville, TN USA; 2 Department of Molecular and Medical Pharmacology, Crump Institute for Molecular Imaging, David Geffen School of Medicine at UCLA, Los Angeles, CA USA; 3 Department of Molecular Biosciences, University of Kansas, Lawrence, KS USA; 4 Stowers Institute for Medical Research, Kansas City, MO USA

## Abstract

**Background:**

Vaults are intriguing ribonucleoprotein assemblies with an unknown function that are conserved among higher eukaryotes. The Pacific coast sea urchin, *Strongylocentrotus purpuratus*, is an invertebrate model organism that is evolutionarily closer to humans than *Drosophila *and *C. elegans*, neither of which possesses vaults. Here we compare the structures of sea urchin and mammalian vaults and analyze the subcellular distribution of vaults during sea urchin embryogenesis.

**Results:**

The sequence of the sea urchin major vault protein (MVP) was assembled from expressed sequence tags and genome traces, and the predicted protein was found to have 64% identity and 81% similarity to rat MVP. Sea urchin MVP includes seven ~50 residue repeats in the N-terminal half of the protein and a predicted coiled coil domain in the C-terminus, as does rat MVP. A cryoelectron microscopy (cryoEM) reconstruction of isolated sea urchin vaults reveals the assembly to have a barrel-shaped external structure that is nearly identical to the rat vault structure. Analysis of the molecular composition of the sea urchin vault indicates that it contains components that may be homologs of the mammalian vault RNA component (vRNA) and protein components (VPARP and TEP1). The sea urchin vault appears to have additional protein components in the molecular weight range of 14–55 kDa that might correspond to molecular contents. Confocal experiments indicate a dramatic relocalization of MVP from the cytoplasm to the nucleus during sea urchin embryogenesis.

**Conclusions:**

These results are suggestive of a role for the vault in delivering macromolecules to the nucleus during development.

## Background

The sea urchin *Strongylocentrotus purpuratus *is an important model system in developmental biology and its genome is currently being sequenced by the Human Genome Sequencing Center at the Baylor College of Medicine under the auspices of the National Human Genome Research Institute (NHGRI). Sea urchin embryos are well suited for biochemical approaches to studying the cell biology of development, as large quantities of eggs can easily be obtained, and their fertilization initiates the synchronous development of optically transparent embryos [1]. Sea urchins occupy an important phylogenetic position as basal deuterostomes, and are thus more closely related to humans than are other invertebrate model organisms such as *Drosophila *and *C. elegans*. In addition, the echinoderm lineage leading to sea urchins diverged from chordates prior to the large scale gene duplication events that occurred early in the evolution of the vertebrates, and because of this many of the genes that are found as multiple paralogues in vertebrates have only a single homolog in sea urchins. Therefore sea urchins provide a system that avoids the problem of functional redundancies between multiple paralogues that often occurs in vertebrates, and serves as a useful comparison for assessing the significance of conserved genes and regulatory linkages within the genome.

Sea urchins cells, like mammalian cells, contain abundant quantities of vaults, which are ~13MDa ribonucleoprotein particles of as yet unknown function [2]. Vaults are barrel-shaped assemblies composed of multiple copies of three proteins and small untranslated RNA molecules, called vRNA. Intriguingly vaults are up-regulated in certain human multidrug-resistant cancer cell lines, although their role in multidrug resistance remains unclear [3-6]. The high level of conservation among higher eukaryotes of both the major vault protein (MVP) sequence and the barrel-shaped vault structure suggests an important cellular role for the vault. Nucleocytoplasmic transport, sequestration of macromolecules, and protection from xenobiotics have all been proposed as possible functions for the vault [4,7-9]. Several recent publications have supported a role for vaults as either a transporter [10,11] or a scaffold protein [12]. Knock-out mice have been produced lacking one each of the three mammalian vault proteins: MVP; the vault poly(ADP-ribose) polymerase (VPARP); and the telomerase associated protein one (TEP1) [13-15]. These knock-out mice appear to be healthy, indicating that if the vault does perform a critical cellular function there must be a redundant or compensatory pathway in the mouse.

Immunofluorescence studies in sea urchins indicate that MVP is present throughout the cytoplasm in cleavage-stage zygotes and in the nucleus in adult somatic cells [16]. Within the nucleus of coelomocytes, the MVP is present in particularly high concentrations in the nucleolus. This is in contrast to localization studies in most other eukaryotic cells, which show MVP as primarily cytoplasmic [17-21]. During isolation from sea urchins, vaults are found to co-purify with both microtubules and ribosomes [22-24]. The sea urchin MVP cell localization and co-purification results led to a hypothesis that vaults may play a role in nucleocytoplasmic transport of ribosomes and/or mRNA [16].

The present study provides a comparison of vaults from sea urchins and rats in terms of their molecular composition and MVP protein sequence, as well as by cryoEM imaging and three-dimensional reconstruction. In addition, images obtained by confocal microscopy are presented that indicate differential localization of MVP during sea urchin embryogenesis, suggesting that the highly conserved ribonucleoprotein vault might play a role during development.

## Results

### Molecular composition of sea urchin vaults and comparison with mammalian vaults

Vaults isolated from mammals, including rats, mice, and humans, are composed of three proteins (MVP, VPARP, and TEP1) and one or more small untranslated RNA molecules, called vRNA. The protein stoichiometry is thought to be 96 copies of MVP with 2–16 copies of VPARP and TEP1 per particle [25]. The human (99 kDa) and rat (96 kDa) homologs of MVP display 91% similarity. In humans the two high molecular weight vault proteins, VPARP and TEP1, have been sequenced and have masses of 193 and 290 kDa, respectively [18,26-28]. vRNA accounts for ~5% of the total mass of the mammalian vault particle. Four human vRNA genes have been identified, encoding vRNAs that range from 86 to 99 bases in length [8,28]. Rat vaults in contrast have only one species of vRNA, 141 bases in length [29], which appears as a ~37 kDa band by SDS-PAGE [25]. All of the mammalian vRNA sequences that have been sequenced thus far show ~80% sequence identity and have similar predicted secondary structures [18]. In rat vaults vRNA can be degraded by a harsh treatment with two RNases leaving the rest of the vault particle structurally intact [25].

**Figure 1:**
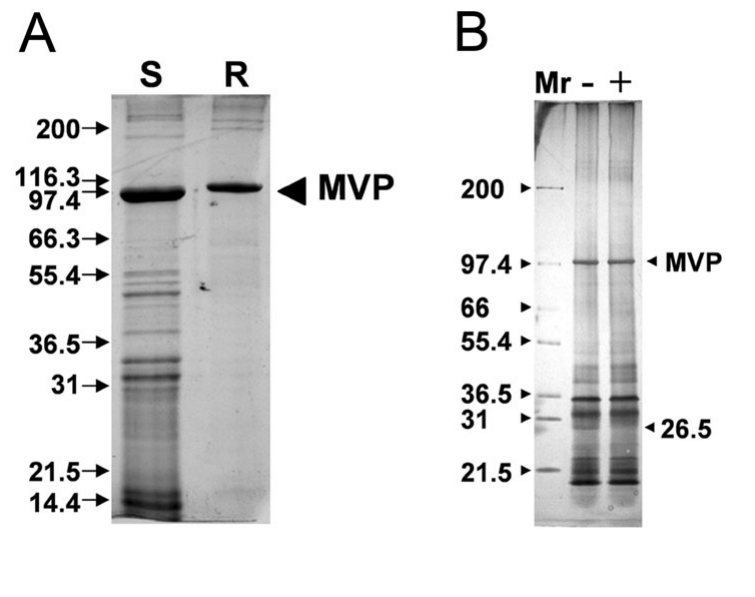
**The molecular composition of the sea urchin vault.**(A) SDS-PAGE analysis (4–16% acrylamide gradient gel) of isolated sea urchin (S) and rat (R) vaults. The band at ~100 kDa in both lanes represents the major vault protein (MVP). (B) SDS-PAGE analysis of sea urchin vaults both without (-) and with (+) RNase treatment. The 26.5 kDa band in the (-) RNase lane, which is missing in the (+) RNase lane, is thought to correspond to the sea urchin vRNA. Mr represents the molecular weight marker lane.

When purified under identical conditions, the molecular composition of the sea urchin vault is more complex than that of the rat vault (Fig. 1A). For both rat and sea urchin vaults, the strongest protein band is that of MVP at ~100 kDa. We have previously demonstrated that polyclonal antiserum generated against the sea urchin ~100 kDa protein has cross-reactivity with the rat vault MVP, as well as with the *Dictyostelium *MVPα and MVPβ vault proteins [16]. SDS-PAGE indicates that the sea urchin vault has two or more high molecular weight components at ~200 kDa, similar to the VPARP and TEP1 bands for rat and mouse vaults [9,30,31]. Searches of the *S. purpuratus *genome identified clear sequence homologs of both VPARP and TEP1 (data not shown). Purified sea urchin vaults also appear to contain vRNA, as demonstrated by the loss of a 26.5 kDa band after RNase treatment (Fig. 1B). We note that the apparent molecular weight of the sea urchin RNA molecule is smaller than that of the rat vault vRNA, 26.5 *vs*. 37 kDa.

What is noticeably different between the sea urchin and rat vaults is the number of protein bands within the molecular weight range of 14 to 55 kDa in the sea urchin vault that are not observed in the rat vault. Since sea urchin vaults were purified in the same manner as rat vaults, we conclude that either the molecular composition of the sea urchin vault is more complex than that of the mammalian vaults, or that the sea urchin vaults have a more varied composition of molecular cargo.

### Amino acid sequence of the sea urchin major vault protein

**Figure 2:**
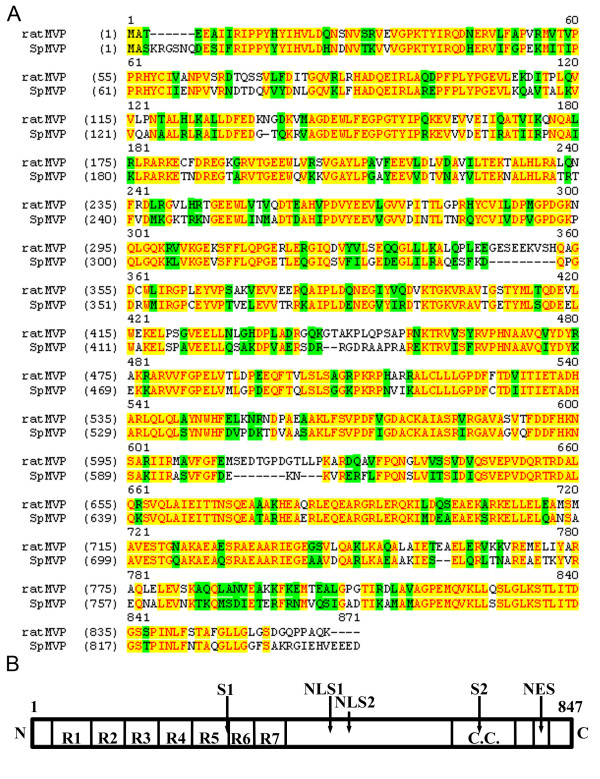
**The sequence of the sea urchin major vault protein (SpMVP). **(A) Amino acid sequence of SpMVP aligned with that of rat MVP. Identical residues are highlighted in yellow, and similar residues are highlighted in green. (B) Bar diagram of SpMVP. The sequence has seven repeats in the N-terminal half of the protein (R1-R7), each repeat consisting of 41 to 62 aa, spanning residues 32–400; and a predicted coiled coil region in the C-terminal half of the sequence (C.C.), residues 663–762. The positions of two possible nuclear localization sequences (NLS1 470-KKAR, and NLS2 498-KPKR), a putative nuclear export sequence (NES, 792–816), and two probable sumoylation sites that are conserved across six species (S1 K308-VKGE, and S2 K707-AKAE) are indicated.

To more fully characterize and identify the sea urchin MVP, we used expressed sequence tags (ESTs) and trace sequences from the *S. purpuratus *genome to assemble the coding sequence of the *SpMVP *gene (see Methods), which is apparently present as a single homolog per haploid genome. The fact that this sequence was found in several blastula-stage ESTs shows that it is expressed in the embryo. The deduced sequence of sea urchin MVP encodes a 95 kDa protein which is slightly smaller than the 96 kDa rat MVP, with 847 *vs*. 861 aa. These two MVP homologs exhibit 64% identity and 81% strong similarity (Fig. 2A). The sea urchin MVP sequence is composed of seven repeats (each 41 to 62 residues) in the N-terminal half of the protein, and a predicted coiled coil region (aa 663–762) in the C-terminal half of the protein (Fig. 2B). The rat MVP sequence has seven similar repeats of unknown function in the N-terminal half and also has a predicted coiled coil region in the C-terminal half [32,33].

In addition to comparing rat and sea urchin MVP sequences, we used pair-wise alignments to compare the amino acid sequences of several other known MVPs, including the two from *Dictyostelium *(Table 1). These comparisons indicate that the MVP sequence is highly conserved between phylogenetically distant species, and that the similarity between sea urchin and rat MVP sequences is comparable to that among distantly related vertebrates, and also to the intraspecific similarity of *Dictyostelium *MVPs.

**Table 1:** Differences between MVP amino acid sequences of various species

	**Rat**	***Xenopus***	**Zebrafish**	**Sea urchin**	***Dictyostelium *A**	***Dictyostelium *B**
**Rat**	0	81 (9.38 %)	120 (13.67 %)	112 (12.86 %)	151 (17.34 %)	161 (18.51 %)
***Xenopus***		0	91 (10.50 %)	90 (10.48 %)	124 (14.44 %)	154 (17.91 %)
**Zebrafish**			0	113 (12.96 %)	148 (16.91 %)	165 (18.97 %)
**Sea Urchin**				0	121 (14.07 %)	157 (18.15 %)
***Dictyostelium *A**					0	137 (15.91 %)
***Dictyostelium *B**						0

Pair-wise sequence alignments of MVPs from each species were used to obtain number of amino acids that are not identical, strongly similar, or weakly similar between each pair of sequences. The numbers in parentheses represent corresponding percent difference. The two *Dictyostelium *MVPs are designated A and B.

The sea urchin MVP sequence was analyzed for potential nuclear localization signals (NLS's) and nuclear export signals (NES's), as well as possible sumoylation sites. Although the PredictNLS server [34] did not find any putative NLS's, visual examination of the sequence resulted identification of two basic regions with three lysine (K) or arginine (R) residues within a four amino acid stretch that are similar to the smallest consensus sequence of the monopartite type NLS [35]. Comparison of the sea urchin MVP sequence with the NES logo defined by la Cour *et al*. [36], indicates that a region of 25 residues near the C-terminus of protein (aa 792 – 816) has a close resemblance to the NES logo. Within this window we find the three most highly conserved leucine (L) residues (L804, L807 and L809); 3 additional residues matching the most favored residue type in the logo; 6 additional residues matching the second or third most favored residue type; and 3 additional residues matching the fourth, fifth of sixth most favored residue type. The Abgents Sumoylation Calculator predicts five highly probable sumoylation sites in order of probability: K308 (VKGE), K731 (LKAE), K707 (AKAE), K736 (AKIE), and K427 (AKDP) within the sea urchin MVP. With the exception of K427, the amino acid sequence surrounding the lysine acceptor residue follow the consensus sequence, ΨKXE, where Ψ represents a hydrophobic residue, K represents the target lysine, X represents any amino acid and E represents glutamic acid [37]. Sequence alignment of MVP homologs from six species showed that two of these five motifs (K308 and K707) are conserved.

**Figure 3:**
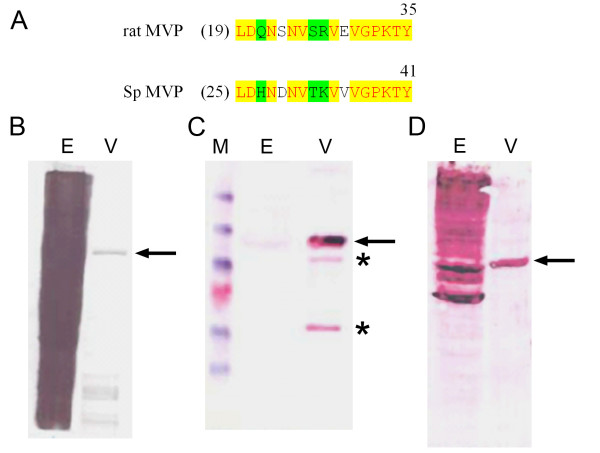
**Western blots of isolated sea urchin vaults. **(A) Peptide amino acid sequence of rat MVP (aa 19–35) aligned with Sp MVP (25–41). Anti-peptide antibodies were generated against the rat MVP sequence shown and affinity-purified as previously described [38]. Twelve of the rat MVP amino acids are conserved in the Sp MVP peptide sequence. (B) 150 μg of sea urchin egg extract proteins (lane E) and 10 μg of purified sea urchin vault proteins (lane V) were separated on this Coomassie-blue stained SDS-8% polyacrylamide mini-gel. The arrow shows the position of the 100 kDa MVP in (B, C and D). (C) Alkaline-phosphatase stained western blot showing the migration of the pre-stained protein ladder (lane M: 176.5, 113.7, 80.9, 63.8 (pink), 49.5, and 8.4 kDa polypeptides). Affinity-purified anti-sea urchin MVP antibodies [16] recognize a 100 kDa polypeptide in egg extracts (lane E) and in purified vault preparations (lane V). Asterisks indicate two bands of approximately 80 kDa and 50 kDa that may be breakdown products of the MVP. (D) Affinity-purified anti-peptide antibodies (anti-LDQN, [38]) bind to the 100 kDa MVP polypeptide in purified sea urchin vaults. This peptide antibody appears to bind non-specifically to a large number of polypeptides in the sea urchin egg extracts.

Epitopes are also conserved between the rat MVP and the sea urchin MVP. Previously, we generated and purified a rabbit polyclonal antibody against the MVP that copurified with sea urchin microtubules [16]. These antibodies were shown to recognize both *Dictyostelium *and rat MVP [16]. In this report, we show that these affinity-purified antibodies also recognize the MVP in purified sea urchin vault preparations (Fig. 3). Two minor bands of ~80 kDa and 50 kDa cross-react with the anti-MVP antibodies indicating that these may be breakdown products of the 100 kDa MVP. In addition to the antibodies generated against the sea urchin MVP, anti-peptide antibodies were generated against a peptide located at the amino terminus of the rat MVP sequence [38]. This sequence is approximately 70% identical in the sea urchin and rat MVP sequence and the affinity-purified anti-peptide antibodies bind to purified sea urchin MVP (Fig. 3). This observation provides experimental support to relate the assembled coding sequence of the *SpMVP *gene to the major protein component of the purified sea urchin vault.

### Electron microscopy and reconstruction of the sea urchin vault

**Figure 4:**
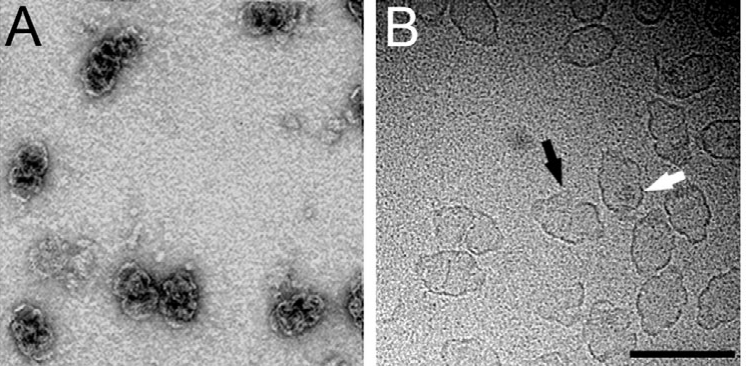
**Negative-stain and cryoEM images of isolated sea urchin vaults. **(A) Negative-stain electron micrograph. (B) Cryoelectron micrograph. The black arrow indicates a vault that is opening at the midsection, and the white arrow indicates the dark molecular contents within another particle. The scale bar represents 1,000 Å. Note that the magnification of the cryoEM image (B) is slightly higher than that of the negative-stain EM image (A).

Negative-stain EM and cryoEM images of isolated sea urchin vaults reveal that they have the same overall morphology as mammalian vaults (Fig. 4). As was noted in cryomicrographs of the rat vault [9], sea urchin vaults are occasionally observed to be 'open' at the midsection. One advantage of cryoEM over negative-stain TEM is that a cryomicrograph is essentially a projection image of all of the density, external and internal, within a macromolecular assembly. In cryomicrographs, both sea urchin and rat vaults appear to have molecular contents of varying mass per particle (Fig. 4B, white arrow and [9]).

**Figure 5:**
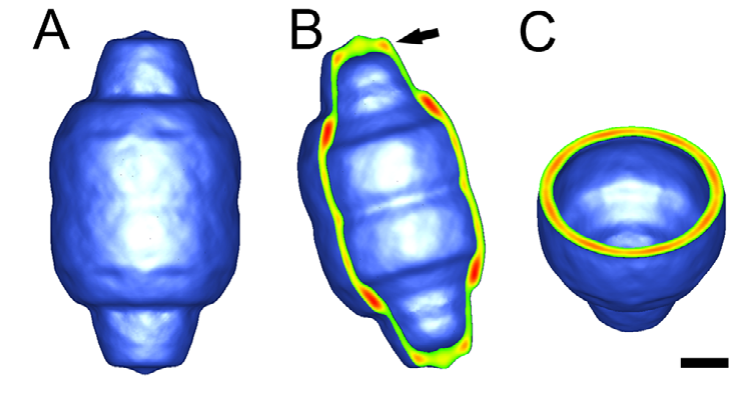
**The sea urchin vault reconstruction at 33 Å resolution. **(A) The full reconstruction, which reveals that the sea urchin vault has essentially the same exterior structure as rat and mouse vaults. (B and C) The reconstruction is shown cropped along two perpendicular axes to reveal the hollow interior. The crop planes are displayed with the strongest density in red and the weakest density in green. Note that the strongest density is in the "shoulder" region at the top and bottom of the central barrel section. The flat portion of one cap is indicated by an arrow in (B). The scale bar represents 100 Å.

In order to characterize the three dimensional structure of the sea urchin vault, a three-dimensional structure was calculated using cryoEM images and single particle reconstruction methods. Only particle images of well-formed, fully closed sea urchin vaults were selected for image processing. Refinement of the cryoEM data set produced a three-dimensional reconstruction at 33 Å resolution (Fig. 5). The exterior shape of the sea urchin vault reconstruction is nearly identical to the shape of the rat vault reconstruction with a central barrel section and two protruding caps [9]. When the sea urchin vault reconstruction is cropped in half the large internal cavity is revealed (Fig. 5B and 5C). As has been noted for the rat vault, the volume of the internal cavity is large enough to enclose an intact ribosome [9].

CryoEM reconstructions of recombinant and tissue derived vaults often show small holes at the cap/barrel junction [9,39]. The sea urchin vault reconstruction does not show these holes, but they are probably obscured by the low resolution of the sea urchin vault structure. Another subtle difference between the sea urchin and rat vault reconstructions is that less density is observed in the flat portion of the sea urchin vault cap (Fig. 5B, arrow). This is the same region of the vault that was identified as the vRNA binding site within the rat vault [25]. It is also a region that tends to be variable between vault reconstructions [39].

### Cellular localization of MVP during sea urchin embryonic development

**Figure 6:**
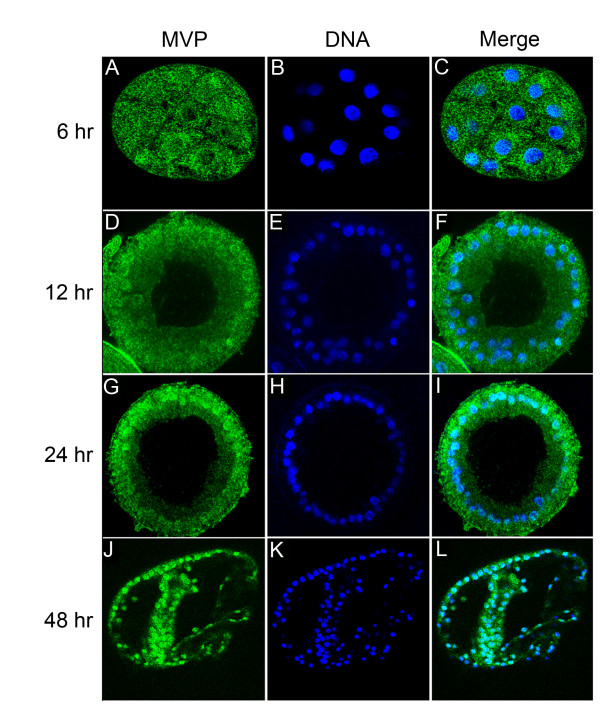
**Confocal images of sea urchin embryos at various developmental stages. **The cellular distribution of MVP is revealed by staining with an affinity-purified polyclonal antiserum against the sea urchin MVP and secondary staining with Oregon Green-conjugated anti-rabbit antibody (A, D, G, J; left column). The nuclei are stained with DAPI (B, E, H, K; middle column). By merging the MVP and DNA images coincident staining is observed as cyan (C, F, I, L; right column). Note that as development proceeds the cellular localization of MVP progressively shifts from cytoplasmic to nuclear.

Earlier immunofluorescence studies indicated that maternal and zygotic MVP is located in the cytoplasm, whereas adult MVP is predominantly nuclear [16]. The subcellular location of MVP during embryogenesis was not determined by immunofluorescence. However, cell fractionation and immunoblot analyses of embryos showed that while total amount of MVP remained constant during embryogenesis, MVP became progressively concentrated in the nuclear fractions as development proceeded from the mesenchyme blastula stage to the larval stage [16]. Here we confirm and extend this result using antibody staining and confocal microscopy to localize MVP subcellularly during embryonic development. The confocal images in Figure 6 demonstrate that sea urchin MVP moves from a largely cytoplasmic distribution in the cleavage stage embryo, to a predominantly nuclear and/or perinuclear location at blastula and gastrula stages.

## Discussion

In this study we used the sea urchin genome to deduce the amino acid sequence of the sea urchin MVP and characterized the structure of the sea urchin vault particle using cryoEM single particle reconstruction methods. It is known that multiple copies of MVP form the recognizable vault structure, as expression of rat MVP in insect cells leads to the assembly of vaults [40]. Given the relatively high (81%) sequence similarity observed between sea urchin and rat MVP, it is not surprising that the exterior sea urchin vault structure is quite similar to that of rat vaults [9,39]. In addition to MVP, the rat vault is composed of the high molecular weight proteins VPARP and TEP1, as well as the small vRNA. The electrophoretic analysis of purified sea urchin vault presented here shows protein bands and one RNA band that might correspond to homologs of the mammalian vault components VPARP, TEP1, and vRNA. Moreover, sequence homologs of VPARP and TEP1 are found in the sea urchin genome.

SDS-PAGE analysis has indicated that the molecular composition of sea urchin vaults is more complex than that of rat vaults. Protein bands in the range of 14 to 55 kDa are observed that might either be additional vault components or macromolecular contents that are present in high copy numbers within the sea urchin vault. One protein in this mass range (~50 kDa) cross-reacts with MVP antisera and is likely to be a breakdown product of MVP. The identities of the additional proteins will be the subject of future investigations. It is tempting to speculate that the difference in protein composition between sea urchin and rat vaults might indicate that sea urchin vaults are transporting or sequestering a larger assortment of macromolecules.

Vaults were originally named because of their similarity to arched cathedral ceilings [41]. This name may be apt in another sense in that the vault might serve to provide a safe enclosure for its molecular contents [2]. It has been postulated that mammalian vaults might open under certain physiological conditions to allow encapsulation or release of their molecular cargo. Sea urchin vaults are occasionally observed to be partially open at the midsection in cryomicrographs. Thus sea urchin vaults, as well as mammalian vaults, might be able to regulate what is contained within the large internal cavity. Both sea urchin and rat vaults [9] appear to have molecular contents in cryomicrographs. However as the features of the contents vary from vault to vault they are inappropriately averaged or "smeared" during the image reconstruction process.

A difference map analysis between cryoEM reconstructions of intact and RNase-treated rat vaults led to the localization of the vRNA to the flat portions of the rat vault caps [25]. The reconstruction of the sea urchin vault reveals a difference in this region of the vault and appears more similar to the RNase-treated rat vault [25]. Although RNase-treatment does not harm the structure of the rat vault, it does appear to affect the sea urchin vault. Several attempts were made to collect negative-stain and cryoEM images of the RNase-treated sea urchin vaults, but no intact vault particles were observed. The difference observed in the cap density and the differing response to RNase-treatment could imply that the vRNA has a different location or a more important structural role in the sea urchin vault than in the rat vault.

The confocal images reveal a progressive enrichment of MVP in the nucleus and perinuclear cytoplasm during sea urchin embryogenesis. Our previous biochemical fractionation studies confirm that the MVP accumulates in the nucleus beginning at the mesenchyme blastula stage [16]. This important stage of development marks the transition between proliferation and differentiation. As a consequence of differentiation the cells are preparing to exit from the rapid cell cycles that followed fertilization. For example, mRNAs which were abundant in the early cleavage stages, such as cyclin mRNA, decrease dramatically at the blastula stage [42]. New post-cleavage stage mRNAs begin to accumulate at blastula stage. We suggest that vaults participate in nucleocytoplasmic transport that accompanies remodeling of nuclear architecture in preparation for terminal cell differentiation [43].

While MVP localization studies in mammalian cells reveal a largely cytoplasmic location, the published staining pattern is consistent with there also being a small amount of nuclear MVP [17,18]. While no classical NLS sequence is found in either the sea urchin or rat MVP, there are basic sequences that might potentially fulfill the requirements of an NLS. The C-terminal region of sea urchin MVP has a region that strongly resembles the NES logo assembled from known NES's [36] and this region is fairly well conserved in MVP from six species. In addition to possible NLS and NES signals, there are several sumoylation signals within the sea urchin MVP.

Sumoylation is a dynamic and reversible process that involves the covalent attachment of the small ubiquitin-like modifier (SUMO) protein to protein substrates. Similar to ubiquination, sumoylation requires four enzymatic steps to attach the carboxyl-terminal glycine of SUMO to the ε-amino group of lysine (reviewed in [44]). However, unlike traditional ubiquination, sumoylation does not mark the protein for degradation. The wide variety of SUMO targets identified to date does not reveal a single function for this protein modification (reviewed in [45]). A variety of transcription factors and cofactors, viral proteins, proteins associated with nuclear architecture and genome surveillance, as well as signal transduction molecules appear to by sumoylated [44]. Importantly for this study, two proteins of the nuclear pore complex, RanGAP1 and Ran-binding protein 2 are both SUMO substrates. Sumoylation of RanGAP1 is important for the nucleocytoplasmic transport of this cytoplasmic nuclear import factor [46-48]. In addition, RanBP2, a docking protein at the cytoplasmic surface of the nuclear protein complex is a SUMO E3 ligase, also known as Nup358 [49-51]. These observations suggest that SUMO plays an important role in nucleocytoplasmic trafficking. In this regard, sumoylation of the major vault protein may regulate the transport of the vault particle through the nuclear pore complex.

Taken together these results suggest that the vault may play a role in delivering macromolecules to the nucleus during embryonic development in sea urchins. A trafficking role has also been proposed for vaults in human cells based on the observations that MVP interacts with the estrogen receptor in MCF-7 breast cancer cells [10] and with the tumor suppressor PTEN in HeLa cells [11]. Estrogen receptor is involved in regulating eukaryotic gene expression and can affect cellular proliferation and differentiation. Estrogen treatment is observed to increase the association of the estrogen receptor with MVP in the nuclei of MCF-7 cells, thus leading to the hypothesis that vaults may be involved in nucleocytoplasmic shuttling of the estrogen receptor and in modulating the effect of steroid hormones. PTEN negatively regulates the phosphoinositide 3-kinase pathway and cell growth. MVP has been shown by a yeast two-hybrid screen to be a dominant PTEN-binding protein. It has been postulated that perhaps the vault serves to mediate the cellular localization PTEN, thus potentially affecting cell growth.

The results presented here suggest that sea urchin vaults contain various macromolecules in addition to the two main vault-associated proteins, VPARP and TEP1. Human MVP is known to interact specifically with the tyrosine phosphatase SHP-2, an enzyme that plays an important role in intracellular signaling [12]. It has been demonstrated using MVP-deficient mouse fibroblasts that MVP helps to support cell survival. MVP is proposed to act as a scaffold protein for SHP-2 and other extracellular-regulated kinases (Erks) and thus facilitate, or somehow modulate, growth factor signaling. Perhaps the varied macromolecular contents of the sea urchin vault include proteins involved in sea urchin growth factor signaling.

## Conclusions

It would seem likely that the sea urchin MVP is transported to the nucleus for a reason. Presumably cells must expend a considerable amount of energy to achieve this differential localization, as MVP is a relatively abundant protein. The MVP concentration in the unfertilized sea urchin egg is on the order of 10 μM, as determined by immunoblotting [16]. This maternal store of MVP is sufficient to assemble 10^7 ^intact vault particles. By comparison, the mature sea urchin egg contains approximately 4 × 10^8 ^ribosomes [52]. Although the function of the vault is not yet clearly delineated, our results on the highly conserved structure, molecular composition, and differential cellular localization of MVP suggest that vaults may be important for nucleocytoplasmic trafficking during embryonic development.

## Methods

### Isolation of sea urchin and rat vaults

Vaults were purified to homogeneity from unfertilized eggs of the Pacific coast sea urchin *Strongylocentrotus purpuratus*. Animals were spawned and the eggs were collected and washed as described [53]. Washed eggs (150 ml) were homogenized with a motor driven Potter-Elvehjem-type tissue grinder in an equal volume of buffer (100 mM PIPES-KOH, pH 7.3, 1 mM MgSO_4_, 4 mM EGTA, 2 mM DTT, 1 mM GTP, 0.2 mM PMSF, 10 μg/ml leupeptin and 1 μg/ml pepstatin A). The homogenate was centrifuged at 18 K rpm (~39,000 g) in a Beckman J20 rotor at 4°C for 45 min. The resulting supernatant fluids were decanted into a 500-ml Erlenmeyer flask at room temperature. A fresh aliquot of GTP was added such that the GTP concentration was increased by 1 mM. The DMSO was added in three aliquots with gentle mixing until a final concentration of 15% (v/v) was reached. Microtubule assembly was promoted by incubating the supernatant fluids in a 24°C water bath for 30–45 min. The microtubules were pelleted by centrifugation at 18K rpm (~39,000 g) in a Beckman J20 rotor at 24°C for 30 min. The supernatant fluids (H1S) were carefully removed and drop-frozen in liquid nitrogen and stored in a -80°C freezer. Microtubules in the pellet were purified by two subsequent cycles of assembly and disassembly as previously described [54] and used for purposes other than the current study.

Sea urchin vaults were purified from the microtubule-depleted supernatant fluids that had been previously frozen in liquid nitrogen. Approximately 150 ml of frozen supernatant (H1S) was thawed and mixed with an equal volume of 50 mM Tris-HCL, pH 7.4, 1.5 mM MgCl_2_, 75 mM NaCl, 1 mM DTT and 1 mM PMSF. This pooled supernatant was used as the starting point for the vault purification. This extract was treated as if it was a rat liver homogenate and all subsequent purification steps were as described for rat liver vaults, including purification on sucrose density gradients and two cesium chloride gradients [9].

Rat vaults were purified from liver by sucrose-density and cesium chloride gradient centrifugation as described by the Rome laboratory [9].

### Molecular composition of sea urchin vaults

Vault samples were analyzed by SDS-PAGE with the discontinuous buffer formulation of Laemmli *et al*., [55] and stained with Coomassie Blue R-250 (Fig. 1A) or silver (Fig. 1B).

### Assembly and analysis of the sea urchin major vault protein sequence

Trace sequences from the *S. purpuratus *genome project [56]were used to assemble the coding sequence of the SpMVP gene. Toward this end the TBLASTN algorithm [57,58] was performed using the rat MVP as a query sequence to search the NCBI trace archive of the sea urchin genome that is now at near 10x coverage. Due to the high level of sequence conservation it was possible to assemble the urchin sequence from the TBLASTN results using the rat MVP as a scaffold. The urchin MVP sequence was assembled in such a way that maximum similarity to the rat scaffold sequence was maintained. The fully assembled SpMVP sequence was then verified by using it to query the NCBI sea urchin EST collection (representing mRNAs expressed in the embryo and larva) by TBLASTN. Except for a small region (A728 to G785) near the C-terminal sequence, the entire SpMVP was covered by *S. purpuratus *ESTs, with the following NCBI accession numbers: BG784484, BG784394, CD305511, CD295832, CD295616, CD304000, CD310423, CD307400, CD305382, CD292179, CD305646, CD333690, CD309547, CD292477, and BG783194. The gap from A728 to G785 was covered by a sequence in the NCBI trace archives (gnl|ti|287010960). The assembled SpMVP sequence has been deposited in the NCBI database under accession number BK005641.

We note that the sea urchin MVP has a molecular weight of 95 kDa, calculated from the protein sequence. Previously the sea urchin MVP was referred to as a 107-kDa polypeptide based on its apparent molecular weight by SDS-PAGE [16]. The program COILS [59] was used to identify a likely coiled coil region in the C-terminal half of the protein (aa 663–762) [60]. The sea urchin MVP sequence was submitted to the PredictNLS server [34,61]. No potential NLS sequences were found by this server. The two possible NLS sequences shown in Figure 2B are based on sequence observation. The sea urchin MVP sequence was compared by hand to the NES logo described by la Cour *et al*. and based on the alignment of 58 high-quality NES's [36]. The sea urchin MVP sequence was submitted to the Abgent Sumoylation Calculator [62]. Five motifs with high probability of being sumoylation sites were identified (scores 0.6889 to 0.9278). Two of these five motifs are conserved among MVP sequences of six species (*Strongy. purp., Homo sapiens, Mus musculus, Rattus norv., Danio rerio*, and *Xenopus laevis*) and are indicated in Figure 2B. The CLUSTALW alignment between sea urchin and rat MVP in Figure 2A was produced by submitting the sequences to the CLUSTALW server [63].

### Negative-stain electron microscopy

Isolated sea urchin vaults were applied to freshly glow-discharged formvar-coated copper grids (Electron Microscopy Sciences). Excess liquid was wicked away and the samples were briefly air dried. Samples were stained with uranyl acetate and observed using a JEOL 1200EXII transmission electron microscope.

### CryoEM imaging and three-dimensional reconstruction

CryoEM grids were prepared using cryogenic plunge freezing methods described previously [9,64]. Digital cryoelectron micrographs were collected on an FEI/Philips CM120 transmission electron microscope with a LaB_6 _filament, a Gatan 626 cryotransfer holder, and a Gatan slow scan CCD camera (1024 × 1024 pixels, YAG scintillator). One hundred and forty-one cryomicrographs were collected with a nominal magnification of 45,000×, and with two different defocus values (-1.0 and -0.6 μm). The pixel size in the cryomicrographs is 4.1 Å on the molecular scale as determined by calibration with a catalase crystal.

The QVIEW software package was used to extract 481 individual vault particle images with a selection box size of 200 × 200 pixels [65]. The initial translation step was performed by cross-correlating each particle image with an 180° rotated version of itself. The IMAGIC software package was used for all 3D image processing steps [66]. The published RNase-treated rat vault reconstruction was used as a search model in the first round of refinement [9]. C8 symmetry was assumed for the sea urchin vault for the first several rounds of refinement, as was used for the intact rat vault reconstruction [9]. After a few rounds of refinement the two ends of the vault appeared nearly identical, and from then on D8 symmetry was imposed, as was done for the RNase-treated rat vault reconstruction [25]. The particles images were corrected for the contrast transfer function (CTF) of the microscope using the CTF equation published by Baker *et al*. [67]. The following CTF parameters were used: Cs = 2 mm, fraction of amplitude contrast = 0.1, and kV = 120. A subset of 409 particle images that agreed best with the rest of the set were selected for the final reconstruction. The resolution was calculated using the Fourier shell correlation method with an elliptical mask applied to remove noise and disordered contents. The resolution was found to be 33 Å by the 0.5 correlation threshold criterion. The sea urchin vault reconstruction is shown filtered to 33 Å resolution and contoured so that the surface appears continuous.

All of the image processing was performed on HP/Digital alpha unix workstations. The graphics representations were produced with the AVS (Advanced Visual Systems, Inc.) software package.

### Antibody staining and confocal microscopy of whole mounted embryos

Staged embryos were fixed in -20°C Methanol for 20 minutes on ice, then washed three times in PBS plus 0.2% Tween-20 (PBST). Embryos were blocked for 30 minutes on ice in PBST plus 5% Bovine Serum Albumin, then incubated with an appropriate dilution of an affinity purified antibody against MVP [16] overnight at 4°C. Following three washes for 5 minutes each in PBST, the embryos were incubated for 1 hour at room temperature in 1 μg/ml Oregon Green 488 goat anti-rabbit antibody. Embryos were washed three times in PBST and stained in PBST with 300 nM DAPI for 10 minutes at room temperature during the second wash. Digital confocal images were collected using a Leica TCS confocal microscope.

## Authors' contributions

PLS directed the cryoEM and three-dimensional image processing as well as helped to draft the manuscript. MM performed the cryomicroscopy and image processing. JL provided technical assistance. CDS performed the antibody staining and confocal imaging of whole-mounted embryos. AJR assembled the SpMVP sequence and performed the sequence alignments used to calculate sequence similarities and differences. JAC directed the research pertaining to vault localization in the embryo, and drafted Figure 6 and the relevant parts of the manuscript. KAS conceived of the study, participated in its design and coordination, purified the sea urchin vaults, and helped to draft the manuscript. All authors read and approved the final manuscript.
